# Determination of 18 Trace Elements in 10 Batches of the Tibetan Medicine Qishiwei Zhenzhu Pills by Direct Inductively Coupled Plasma-Mass Spectrometry

**DOI:** 10.1155/2022/8548378

**Published:** 2022-01-13

**Authors:** Ke Fu, Yinglian Song, Dewei Zhang, Min Xu, Ruixia Wu, Xueqing Xiong, Xianwu Liu, Lei Wu, Ya Guo, You Zhou, Xiaoli Li, Zhang Wang

**Affiliations:** ^1^College of Pharmacy, Chengdu University of Traditional Chinese Medicine, Chengdu 611137, China; ^2^Wanzhou Institute for Drug and Food Control, Chongqing 404000, China; ^3^College of Ethnomedicine, Chengdu University of Traditional Chinese Medicine, Chengdu 611137, China

## Abstract

Qishiwei Zhenzhu pills (QSW) was first recorded in the Tibetan medicine classic *Si Bu Yi Dian* and has been used to treat “Baimai” disease, stroke, paralysis, hemiplegia, cerebral hemorrhage, and other diseases till today. This prescription contains more than 70 medicines including myrobalan, pearl, agate, opal, bezoar, coral, musk, gold, silver, and a mineral mixture Zuotai. As a result, QSW contains a large amount of mercury, copper, lead, and other trace elements. The aim of this study was to determine the 18 trace elements (lithium, beryllium, scandium, vanadium, chromium, manganese, cobalt, nickel, copper, arsenic, strontium, argentum, cadmium, cesium, barium, lead, aurum, and mercury) in 10 batches of QSW produced by 5 pharmaceutical companies (Ganlu Tibetan Medicine Co., Ltd. has 6 different batches) by direct inductively coupled plasma-mass spectrometry (ICP-MS). ICP-MS is a rapid, sensitive, accurate methodology allowing the determination of 18 elements simultaneously. The results showed that each element had an excellent linear relationship in the corresponding mass concentration range. The results showed that the rank order of the elements in QSW was copper > mercury > lead from high to low, with the mass fraction higher than 6000 *μ*g/kg; the mass fractions of argentum, arsenic, manganese, aurum, strontium, barium, chromium, and nickel were in the range of 33–1034 *μ*g/kg; and the mass fractions of vanadium, cobalt, lithium, beryllium, cadmium, scandium, and cesium were lower than 10 *μ*g/kg. The reproducibility from the same manufacturer (Tibet Ganlu Tibetan Medicine Co., Ltd.) was relatively high; however, the element amounts among 5 manufacturers were different, which could affect the efficacy and toxicity of QSW. All in all, ICP-MS can be used as an effective tool for the analysis of trace elements in QSW and standard quality control needs to be enforced across different manufactures.

## 1. Introduction

Qishiwei Zhenzhu pills (QSW), a Tibetan medicine རཏྣ་བསམ་འཕེལ།, is also known as Ranna Sangpei. It was first recorded in the great classical work of Tibetan medicine, *Si Bu Yi Dian*. QSW has the functions of tranquilizing mind, activating “meridians and collaterals”, and harmonizing “Qi and blood.” It is mainly used to treat “Baimai” disease and “Longxue” disorder, stroke, paralysis, hemiplegia, epilepsy, cerebral hemorrhage, and other diseases and is listed in *the 2020 edition of Pharmacopoeia of China* [1]. QSW contains more than 70 medicines including herbs, animal products, and minerals [2]. QSW is effective experimentally against vascular dementia in rats [3] and protects against lipopolysaccharide plus 1-methyl-4-phenyl-1,2,3,6-tetrahydropyridine-induced chronic neuroinflammation and dopaminergic neuron loss in mice [4]. We have demonstrated that QSW is effective against cerebral ischemia/reperfusion injury in rats [5, 6]. Furthermore, QSW can increase the ratio of Bcl-2/Bax, increase the levels of superoxide dismutase and catalase, reduce the levels of malondialdehyde, neurogenic specific olefinol enzyme and S-100*β* protein, alleviate lipid peroxidation injury, and downregulate the expression of caspase-3 protein to achieve the protective effect on brain tissue [7–9]. Besides, QSW at therapeutic doses appears to be safe in acute toxicity studies in rats [10] and does not affect hepatic cytochrome P450 in mice [11].

In the preliminary work, we used high-performance liquid chromatography to simultaneously determine the six components in QSW, including gallic acid, corilagin, agarwood, ellagic acid, crocin I, and crocin II, in order to reveal the plant-based active components in QSW [12]. We have also used gas chromatography-mass spectrometry to profile the fat-soluble and volatile components in QSW and found that the fat-soluble and volatile components are the active components in QSW [5, 13]. Besides, we have recently used liquid chromatography-mass spectrometry to identify 42 chemical components in QSW, including 11 triterpenoids, 10 flavonoids, 8 organic acids and their derivatives, 4 diterpenoids, 4 tannins, 2 steroids, and 3 other components. It is worth mentioning that chlorogenic acid, ellagic acid, luteolin, cholic acid, and glycyrrhizin are detected in QSW for the first time [14]. A large number of trace elements contained in QSW, as its potential active components, are mainly derived from minerals. Therefore, in addition to botanical components, the mineral (such as pearl, agate, opal, gold, silver, and a mineral mixture Zuotai; Table 1) contained in QSW cannot be ignored. For example, Zuotai (made mainly by processing mercury) is the main ingredient of many precious Tibetan medicine preparations including QSW. Therefore, the determination of the content of mercury and other trace elements in QSW has become an urgent task at present as mercury is a concerned toxic heavy metal.

Inductively coupled plasma-mass spectrometry (ICP-MS) is an advanced technology to analyze more than 70 elements in a solution, and the linear dynamic range can reach 9 orders of magnitude [15]. It has been widely used in the analysis of trace and ultra-trace inorganic elements in medicine, food, and other industries due to its high sensitivity, simultaneous determination of multiple elements, and strong anti-interference ability [16–18]. Therefore, ICP-MS is undoubtedly a very effective method for in vitro quantitative analysis of heavy metals in QSW and traditional drugs. Besides, when QSW or traditional drugs are absorbed by the human body, a series of changes will occur, such as changes in the valence of heavy metals. ICP-MS in conjunction with other instruments for detecting element valence is its better advantage. For examples, Chen et al. established a method for the determination of soluble arsenic and its forms in realgar Chinese patent medicines by biomimetic extraction high-performance liquid chromatography (HPLC)-ICP-MS [19]. Jin et al. used HPLC-ICP-MS to determine the morphological changes of five arsenic species in *Niuhuang Jiedu* tablets, such as betaine, dimethyl arsine, trivalent arsenic, monomethyl arsine, and pentavalent arsenic, so as to make the study of heavy metal elements in *Niuhuang Jiedu* tablets more in-depth and detailed [20].

We have used ICP-MS to determine the absorption, distribution, and excretion of 18 elements in cerebral ischemia-reperfusion rats treated with QSW [21], highlighting the retention of minerals in the gut as a basis of gut-microbiota-brain axis for QSW-induced neuroprotection [5]. But it is worth noting that QSW is granted in the treatment of various diseases [1] and produced by at least 5 pharmaceuticals. Batch-to-batch variation, especially elements/minerals variations, could affect the pharmacological efficacy and toxicity potential of QSW in the treatment of diseases. Quality control in complementary and alternative medicines is needed [22, 23]. Therefore, the aim of the current study was to determine the 18 elements in 10 batches of QSW produced by 5 pharmaceuticals via ICP-MS. The research results can provide a scientific basis for the quality control and pharmacology as well as toxicology of Tibetan medicine.

## 2. Materials and Methods

### 2.1. Reagents and Chemicals

All solutions were prepared with deionized water provided by a water purification system (18 MΩ; Shengdeli Ultra-Pure Water System, Chongqing, China). Nitric acid (HNO_3_, 65%, Suprapur) and hydrogen peroxide (H_2_O_2_, 50%, Suprapur) were obtained from Chongqing Chuandong Chemical Industry Group Co., Ltd. (Chongqing, China). Tuning solutions (1.0 *μ*g/L of barium (Ba), cerium, cobalt (Co), indium (In), lithium (Li), uranium) were purchased from Thermo Fisher Scientific (USA). Internal standards stock solution (1000 *μ*g/mL of rhodium (Rh), In, rhenium (Re), bismuth (Bi)) and gold standards stock solution (1000 *μ*g/mL) were purchased from National Nonferrous Metals and Electronic Materials Analysis and Testing Center (Beijing, China). 1000 *μ*g/mL of reference standard solutions for Li (GSB04-1734-2004), beryllium (Be, GSB04-1718-2004), scandium (Sc, GSB 04-1750-2004), vanadium (V, GSB04-1759-2004), chromium (Cr, GSB04-1723-2004a), manganese (Mn, GSB04-1736-2004), Co (GSB04-1722-2004), nickel (Ni, GSB04-1740-2004), copper (Cu, GSB04-1725-2004), arsenic (As, GSB04-1714-2004), strontium (Sr, GSB 04-1754-2004), argentum (Ag, GSB04-1712-2004), cadmium (Cd, GSB04-1721-2004), cesium (Cs, GSB04-1724-2004), Ba (GSB04-1717-2004), aurum (Au, GSB04-1715-2004), mercury (Hg, GSB04-1729-2004), and lead (Pb, GSB04-1742-2004) were purchased from National Nonferrous Metals and Electronic Materials Analysis and Testing Center (Beijing, China).

QSW from 5 manufacturers in 10 batches were collected (Table 2), which were the most popular brands in the Chinese market.

### 2.2. Preparation of Reference Standard Solutions and Internal Standards Stock Solution

The reference standard solutions containing 18 kinds of metal elements to be measured, such as Li, Be, Sc, V, Cr, Mn, and Co, are precisely measured, and when it is used, it is diluted with 10% nitric acid into a mixed reference standard solution of various element series mass concentration: Li, Be, Sc, V, Co, Cd, Cs (0, 0.5, 2.0, 10, 25, 50 *μ*g/L); Cr, Mn, Ni, Cu, As, Sr, Ag, Ba, Au, Pb (0, 0.5, 2.0, 10, 25, 50 mg/L); Hg (0, 20, 50, 100, 150, 200 *μ*g/L). The reference standard solution of mercury is separately configured, and the other 17 elements are configured as a mixed reference standard solution. All reference standard solutions are in a constant volume of 50 ml.

The internal standards stock solution containing Rh, Re, In, and Bi was diluted with 10% nitric acid to form a mixed internal standards stock solution with a concentration of 10 *μ*g/mL, which was used to correct the interference caused by the matrix effect and ensure the accuracy of the determination results.

### 2.3. Preparation of Gold Standards Stock Solution

A suitable amount of gold standards stock solution is diluted with 10% nitric acid to 10 *μ*g/mL and stored in a refrigerator. (When Hg is added, it is easy to produce the memory effect and adsorption effect, which affect the accuracy of the determination results. Hence, 20 *μ*L of gold standard solution was added to stabilize.)

### 2.4. Sample Preparation

QSW samples were digested by the microwave digestion system (Matching PTFE Tank, Milestone ETHOS A; Shenzhen Huashengda Instrument Equipment Co., Ltd., Shenzhen, China). 10 batches of QSW were wrapped and crushed with filter paper (no grinding with mortar to prevent the introduction of heavy metals); then 0.0500 g of the above-mentioned tablets powder was accurately weighed in a microwave digestion tube, into which 10 mL of Suprapur® concentrated nitric acid (65%) solution and 0.2 mL of hydrogen peroxide solution (50%) were added; after gently shaking, tighten the cover of the digestion tube, place the digestion tube evenly on the turntable in the microwave oven, close the door of the oven, and input the microwave heating program (Table 3). The digestion time is 60 minutes. After digestion, the volume was adjusted to 50 mL with ultra-pure water, Hg is easy to be adsorbed, and then 0.1 mL of the mixture was added into a 50 mL volumetric bottle with gold standard solution to stabilize.

### 2.5. Operating Conditions of ICP-MS

Multielement detection was carried out using an ICP-MS system (ICAP-Q Series; Thermo Fisher Scientific, USA). The parameters of ICP-MS system are shown in Table 4.

### 2.6. Linearity and Limit of Quantitation (LOQ)

The multielement mixed standard solution under “Section 2.2” was injected into the instrument at the same time with the internal standard solution and determined in turn; the average measured values of the three readings of each mass concentration are ordinate (*Y*), and the corresponding mass concentration of the standard solution of the corresponding elements is abscissa (*X*). In addition, the blank solution was prepared and determined for 11 consecutive times, and the LOQ of different trace elements were determined by dividing the standard deviation of the response value by the slope of the standard curve of the corresponding elements.

### 2.7. Precision and Repeatability

The contents of 18 trace elements Li, Be, Sc, V, Cr, Mn, Co, Ni, Cu, As, Sr, Ag, Cd, Cs, Ba, Pb, Au, and Hg were determined for 6 consecutive injections, and the response values of each element were calculated. Besides, QSW (batch number 17156A) was selected as the sample to prepare six sample solutions in parallel, and the response values of each element were calculated.

### 2.8. Accuracy

QSW (batch number 17156A) was selected as the samples for investigation; about 0.0250 g sample was precisely weighed, precision addition of appropriate standard element solution (about 100% level of known content). According to the preparation method of sample solution under “Section 2.4” and ICP-MS working parameters under “Section 2.5”, the recovery rate of 18 elements was calculated.

### 2.9. Statistical Analysis

The experimental data were processed by SPSS 21.0 software, and the measurement data were expressed as mean ± SD.

## 3. Results

### 3.1. Linearity and Limit of Quantitation (LOQ)

The standard curve was drawn, linear regression was carried out, and the regression equations of 18 trace elements were obtained. In addition, the LOQ of the 18 trace elements all met the analysis requirements (Table 5).

### 3.2. Precision and Repeatability

The precision of 18 elements was good, which can meet the requirements of analysis. Besides, %RSD values of the 18 elements were less than 5%, indicating good repeatability (Table 5).

### 3.3. Accuracy

The recovery rate of 18 elements was calculated (Table 5). The average recovery of 18 elements was 95.10%–111.13% (%RSD was 2.13%–4.24%).

### 3.4. Results of 18 Trace Elements in 10 Batches of QSW

**Figure 1:**
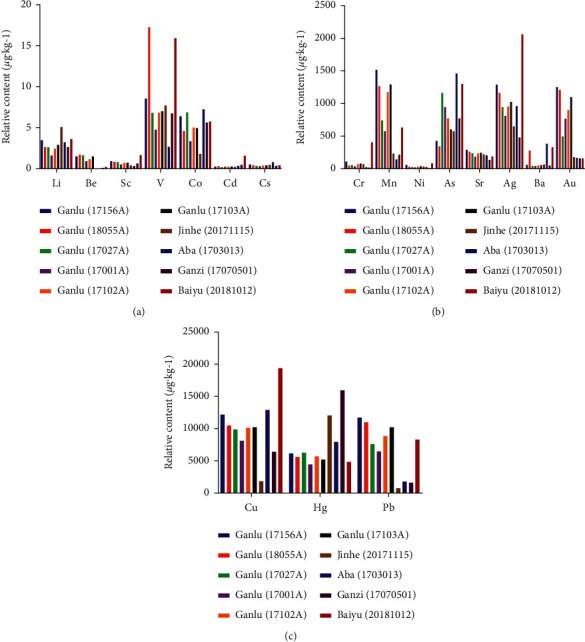
The content of 18 elements in 10 batches of QSW.

**Figure 2:**
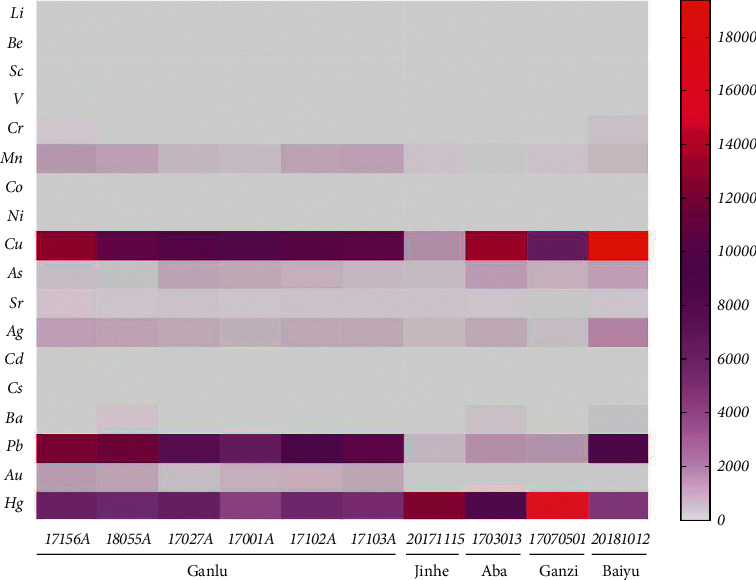
The heat map of determination results of trace elements in 10 batches of QSW. (Note: the redder the color, the higher the content; the grayer the color, the lower the content. For example, the figure shows that the content of Hg in Ganzi (17070501) and Cu in Baiyu (20181012) is very high.)

The contents of 18 trace elements in 10 batches of QSW are shown in Table 6 and Figure 1. The heat map of the determination results of trace elements in 10 batches of QSW is shown in Figure 2. Overall, the average element contents of 10 batches of QSW were in the order of Cu > Hg > Pb from high to low, with the mass fraction higher than 6000 *μ*g/kg; the mass fractions of Ag, As, Mn, Au Sr, Ba, Cr, and Ni were in the range of 33–1034 *μ*g/kg; and the mass fractions of V, Co, Li, Be, Cd, Sc, and Cs were lower than 10 *μ*g/kg.

### 3.5. Visual Analysis

**Figure 3:**
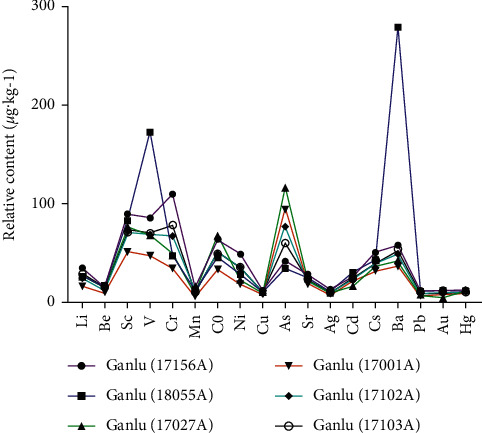
Intuitive analysis of the determination of trace elements in the samples of QSW. (The same manufacturers have similar peak shapes. Among them, only the contents of V and Ba in batch 18055A are slightly different from other batches.) Expansion 10 times: Li, Be, V Co. Expansion 100 times: Sc, Cd, Cs. Reduction 10 times: As, Sr. Reduction 100 times: Mn, Ag, Au. Reduction 1000 times: Cu, Hg, Pb.

**Figure 4:**
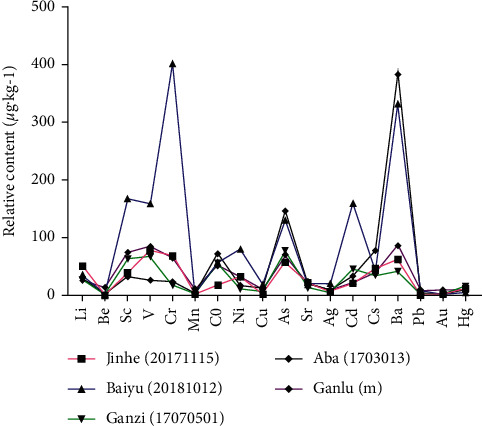
Intuitive analysis of the determination of trace elements in samples of QSW. (The peak shapes of different manufacturers are different, indicating that the content of trace elements in QSW of different manufacturers is quite different.) Ganlu (m) represents the average value of Ganlu's 6 batches of QSW. Expansion 10 times: Li, Be, V Co. Expansion 100 times: Sc, Cd, Cs. Reduction 10 times: As, Sr. Reduction 100 times: Mn, Ag, Au. Reduction 1000 times: Cu, Hg, Pb.

According to the test results of trace elements, 18 trace elements were screened out and mass distribution curves were made according to their atomic number. In order to facilitate comparison, some trace elements with great difference in quantity are enlarged or reduced to the same order of magnitude at the same time of measurement (expansion 10 times: Li, Be, V, Co; expansion 100 times: Sc, Cd, Cs; reduction 10 times: As, Sr; reduction 100 times: Mn, Ag, Au; reduction 1000 times: Cu, Hg, Pb). QSW samples from Ganlu Pharmaceuticals have similar peak shapes. Among them, only the contents of V and Ba in batch 18055A were slightly different from other batches (Figure 3). However, different manufacturers have different peak shapes, with QSW from Baiyu had higher Sc, V, Cr, As, Cd, and Ba and QSW from Aba had As and Ba. Besides, the contents of trace elements in QSW produced by Jinhe and Ganzi were similar to that produced by Ganlu (Figure 4). The results imply that the contents of trace elements in QSW could be different from different manufacturers.

## 4. Discussion and Conclusion

In this article, we have established a sensitive, fast, and efficient ICP-MS method for the determination of trace elements in QSW. The contents of 18 trace elements (Li, Be, Sc, V, Cr, Mn, Co, Ni, Cu, As, Sr, Ag, Cd, Cs, Ba, Pb, Au, Hg) in 10 batches of QSW from 5 pharmaceuticals were determined, and batch-to-batch variations were revealed. The results point to the importance of quality control in the production of QSW and provide an element reference for the quality control of QSW, a famous Tibetan medicine listed in *the 2020 edition of Pharmacopoeia of China* [1].

Traditional Tibetan medicine preparations have added minerals and heavy metal elements as active components. Many valuable Tibetan medicines contain Zuotai as the main ingredient. For example, QSW, *Ershiwuwei Zhenzhu Pills, Renqing Changjue* pills, and *Renqing Mangjue* pills all contain Zuotai and have been included in the Chinese Pharmacopoeia [1]. Zuotai is mainly made of mercury [24]. Its main components are beta-HgS and sulfur [25], and it is insoluble in acid [26]. It is not used alone but as an addition to Tibetan medicine formulae in clinic to increase the curative effect, reduce side effects, invigorate the “spleen,” and nourish and strengthen the body [27]. The results of this study show that the average content of Hg in QSW of various manufacturers is extremely high, among which the highest can reach 15940 *μ*g/kg, with the average amount of 7598 *μ*g/kg, the 2^nd^ highest element in QSW. Mercury is a toxic heavy metal of public safety concern, but mercury sulfides are frequently included in Indian Ayurvedic medicine [28], Tibetan medicines [29], and Chinese medicines [30]. The chemical form of Hg (HgS vs HgCl_2_) makes a big difference. Another important factor is “dose differentiates a poison from a remedy.” The amount of Hg contained in QSW varies 3.6-fold (4397–15940 *μ*g/kg) from batch to batch; a low amount of HgS could compromise it therapeutic effects, but a high amount of Hg could increase its risk. Thus, quality control in evaluating different batch products of QSW for Hg content is important.

Another toxic heavy metal of concern is lead (Pb). Pb-based traditional herbometallic preparations are also widely used in Ayurveda [31], Tibetan medicines, and Chinese medicines [29], but with increasing public alert [32, 33]. Although the chemical forms of Pb used in traditional medicines (PbS, PbO) are different from environmental Pb compounds (lead acetate, etc.) [29], the amount of lead contained in traditional medicines still alarms the public. For example, overdose of *Mahayograj Guggulu* is the most common cause responsible for Pb intoxication cases from traditional Indian medicines [32]. The amount of Pb contained in QSW varies 16-fold (727–11690 *μ*g/kg); a low amount of PbS may reduce efficacy of QSW, but a high amount of Pb definitely represents a risk to human health [34]. Considering 16-fold variations in Pb content in QSW, the quality control of QSW for Pb content is very important.

Copper (Cu) is an essential element for human body; however, excess Cu is also toxic [35]. In the Tibetan medicine “*Renqing Changjue*”, changes in element concentrations, including Cu, in biofluids and tissues were examined, and the precautions with long-term administration are pointed out [36]. The amount of Cu was the highest among 18 elements in QSW and varied 10-fold (1807–19430 *μ*g/kg), similar to the copper content variation (10-fold) in other traditional medicines [37]. A low amount of CuS could compromise its therapeutic effects, but a high amount of Cu could be toxic, especially for long-term administration. Thus, quality control in products of QSW for Cu content is also important.

Manganese (Mn) is an essential metal that is required as a cofactor for many enzymes and is necessary for optimal biological function; however, Mn overexposure is associated with neurotoxicity [38]. Many Tibetan medicines contain Mn and other trace elements to exert anti-anoxic effects [39]; on the other hand, inappropriate overexposure to Mn from traditional medicine practice caused neurotoxicity [40, 41]. The amount of Mn in QSW ranged from 141 to 1512 *μ*g/kg, with a 10-fold variation in different batches of QSW. Insufficient Mn may reduce QSW therapeutic effects, but a high amount of Mn could be toxic, especially to the brain [38]. Thus, quality control of QSW for Mn content is important.

The rank orders of the 18 elements contained in QSW (mean values in *μ*g/kg) were Cu (10160), Hg (7398), Pb (6814), followed by Ag (1034), As (835), Mn (776), Au (637), Sr (217), Ba (133), Cr (90), and Ni (33). The elements with trace amounts were V (8.426), Co (5.148), Li (3.019), Be (0.856), Sc (0.746), Cs (0.443), and Cd (0.400). Due to different manufacturers of QSW, some of the trace elements fluctuate greatly. In addition to the above discussed major metals with toxicological significance (Hg, Pb, Cu, and Mn), batch-to-batch variations of other elements with appreciable amounts were Ag (2.7-fold), As (3.9-fold), Au (8.1-fold), Ba (6.7-fold), Sr (2.2-fold), Cr (12-fold), and Ni (5-fold). Arsenic is a major metalloid of toxicological significance affecting human health and can be derived from traditional medicines [30, 42], and its content from Aba (1461 *μ*g/kg) was 4 times higher than that from Ganlu 18055A (342 *μ*g/kg). Even for elements with trace amount, such variations could not be ignored. For example, Cd is an accumulative, highly toxic heavy metal, which can also be derived from traditional medicines [33]. In QSW, it had a smallest content but varied 9.6-fold across batches, and Cd from Baiyu (1.595 *μ*g/kg) was 4–10 times higher than that from other batches; such variations should not be ignored. Thus, the contents of these elements in QSW and their batch-to-batch variations could influence their biological functions and also affect their toxicity.

It should be noted that the 6 batches of QSW from Ganlu Tibetan Pharmaceuticals were relatively stable with less batch-to-batch variations compared with other 4 batches from Jinhe, Aba, Ganzi, and Baiyu pharmaceuticals. For example, Cu contents from 6 Ganlu batches ranged from 8616 to 12155 *μ*g/kg, only a 1.4-fold variation, but Cu from Baiyu (19440 *μ*g/kg) was 11.7-fold higher than that of Jinhe (1807 *μ*g/kg); Hg contents from 6 Ganlu batches ranged from 4398 to 6267 *μ*g/kg, only a 1.4-fold variation, but Hg from Ganzi (15734 *μ*g/kg) was 2.8 times higher than that from Ganlu (5545 *μ*g/kg). Pb from 6 Ganlu batches ranged from 6416 to 11690 *μ*g/kg, only a 1.8-fold variation, but Pb from Jinhe (727 *μ*g/kg) was 12.8 times lower than that from Ganlu (9289 *μ*g/kg). Mn from 6 Ganlu batches ranged from 571 to 1512 *μ*g/kg, with 2.6-fold variation, but Mn from Aba (141 *μ*g/kg) was 7.7 times lower than that from Ganlu (1092 *μ*g/kg). Thus, to enforce the same quality control among different pharmaceuticals are necessary.

In conclusion, the present study used ICP-MS to simultaneously determine 18 elements in QSW from 10 batches and analyzed batch-to-batch variations from 5 pharmaceuticals, and the biological and toxicological significance of such batch-to-batch variations were discussed, highlighting the importance for quality control of producing traditional medicines such as QSW.

## Figures and Tables

**Table 1:** Minerals in QSW.

The type of components	Pinyin (Chinese name)	English name
**Mineral and gemstone medicine**	Zhenzhu	Pearl
	Jiuyanshi	Nine's eye
	Shanhu	Coral
	Songshi	Tophus
	Maoyanshi	Cat's eye
	Manao	Agate
	Qingjinshi	Lapis lazuli
	Lanbaoshi	Sapphire

**Metal medicine**	Jin	Gold
	Yin	Silver
	Tong	Copper
	Tie	Iron

**Mixture**	Zuotai	——

The above components are most likely to be the main source of trace elements in QSW.

**Table 2:** Production enterprise, batch number, and approval number of QSW.

No.	Manufacturing enterprise	Batch number	Approval number
1	Ganlu Tibetan Medicine Co., Ltd.	17156A	SFDA approval number: Z54020062
2		18055A	
3		17027A	
4		17001A	
5		17102A	
6		17103A	
7	Jinhe Tibetan Medicine Co., Ltd.	20171115	SFDA approval number: Z63020062
8	Sichuan Aba Tibetan Medical Hospital	1703013	Z20060418
9	Sichuan Ganzi Tibetan Medical Hospital	17070501	Z20060302
10	Sichuan Baiyu Tibetan Medical Research Institute	20181012	Z20061067

**Table 3:** Microwave digestion steps.

No.	Steps
Step 1	Temperature rises to 120°C within 15 min and maintains for 5 min at 1600 W power.
Step 2	Temperature rises to 150°C within 7 min and maintains for 7 min at 1600 W power.
Step 3	Temperature rises to 190°C within 7 min and maintains for 15 min at 1600 W power.

**Table 4:** ICP-MS parameters.

ICP-MS system	Working parameters
RF power	1.3 kW
Carrier gas velocity	1.14 L/min
Sampling depth	6.8 mm
Auxiliary gas flow rate	0.9 L/min
Residence time per point	20 ms
Vertical position of rectangular tube	1.8 mm
Horizontal position of rectangular tube	0.6 mm
Atomizer	Babington
Atomization chamber flow rate	0.98 L/min
Atomization chamber pressure	2.85 × 10^5^ Pa

**Table 5:** Linearity, LOQ, repeatability, precision, and recovery values for the elements.

Elements	Linear equation	*R* ^2^	BEC (ng·mL^−1^)	Linear range (*μ*g·L^−1^)	LOQ (ng·mL^−1^)	Repeatability (%)	Precision (%)	Average recovery	Recovery (%)
**Li**	*Y* = 183.452*X* + 30.010	0.9997	0.164	0∼50	0.0111	3.24	3.92	95.97	3.77
**Be**	*Y* = 120.419*X* + 1.646	0.9997	0.014	0∼50	0.0710	3.45	4.40	100.82	4.24
**Sc**	*Y* = 3568.724*X* + 38.237	0.9999	0.011	0∼50	0.0058	3.64	2.03	105.03	2.57
**V**	*Y* = 11335.341*X* + 785.694	0.9999	0.069	0∼50	0.0186	1.43	2.72	102.21	4.17
**Cr**	*Y* = 18508.555*X* + 9806.206	0.9998	0.530	0∼50000	0.0445	4.33	1.56	99.50	3.40
**Mn**	*Y* = 8186.298*X* + 5839.477	0.9999	0.713	0∼50000	0.0631	4.44	1.26	99.60	3.60
**Co**	*Y* = 33478.709*X* + 423.562	0.9998	0.013	0∼50	0.0030	4.05	2.35	96.56	3.81
**Ni**	*Y* = 8849.339*X* + 2884.817	0.9999	0.326	0∼50000	0.0611	1.66	2.52	104.69	2.13
**Cu**	*Y* = 23141.640*X* + 27031.196	0.9992	1.168	0∼50000	0.0769	4.32	1.39	99.31	3.73
**As**	*Y* = 1441.687*X* + 193.154	0.9999	0.134	0∼50000	0.0709	1.97	1.54	106.64	3.03
**Sr**	*Y* = 10750.883*X* + 18797.632	0.9990	1.748	0∼50000	0.0825	4.19	2.52	111.09	2.95
**Ag**	*Y* = 56823.927*X* + 172271.407	0.9882	3.032	0∼50000	0.2512	1.60	1.68	111.13	3.58
**Cd**	*Y* = 8600.838*X* + 114.995	0.9998	0.013	0∼50	0.0108	3.65	3.27	98.93	4.31
**Cs**	*Y* = 42739.545*X* + 791.782	0.9995	0.019	0∼50	0.0026	3.09	2.58	97.47	3.92
**Ba**	*Y* = 8843.577*X* + 38308.402	0.9993	4.332	0∼50000	0.1084	4.10	2.03	95.10	3.43
**Au**	*Y* = 66686.274*X* + 38970.881	0.9999	0.584	0∼50000	0.0517	4.31	3.82	99.96	3.22
**Hg**	*Y* = 20700.587*X* + 1037.767	0.9989	0.050	0∼200	0.0101	2.72	1.95	101.97	3.86
**Pb**	*Y* = 169100.063*X* + 223195.948	0.9996	1.320	0∼50000	0.1003	4.33	4.18	96.45	3.39

The elements are arranged in the order of their molecular weight.

**Table 6:** Results of 18 trace elements in 10 batches of QSW (*μ*g·kg^−1^).

Elements	Content (*μ*g·kg^−1^)										
	Ganlu						Jinhe	Aba	Ganzi	Baiyu	Mean ± SD
	17156A	18055A	17027A	17001A	17102A	17103A	20171115	1703013	17070501	20181012	
**Li**	3.472	2.635	2.633	1.603	2.431	2.893	5.067	3.210	2.633	3.613	3.019 ± 0.919
**Be**	1.472	1.715	1.627	0.9080	1.118	1.504	——	——	0.07500	0.2260	0.8650 ± 0.7210
**Sc**	0.8980	0.8280	0.7690	0.5140	0.7070	0.7110	0.3920	0.3230	0.6340	1.680	0.7460 ± 0.3770
**V**	8.566	17.26	6.766	4.742	6.819	7.025	7.748	2.665	6.751	15.92	8.426 ± 4.613
**Cr**	109.7	47.45	49.89	34.39	67.48	78.20	68.33	23.55	17.64	401.65	89.83 ± 112.9
**Mn**	1512	1267	737.4	571.4	1174	1293	227.5	141.4	214.1	629.1	776.7 ± 504.7
**Co**	6.367	4.573	6.857	3.326	5.044	4.941	1.771	7.249	5.622	5.733	5.148 ± 1.650
**Ni**	48.87	28.30	22.77	18.18	35.49	35.64	32.55	17.31	10.60	80.26	33.00 ± 19.98
**Cu**	12160	10506	9875	8162	10119	10240	1807	12897	6396	19436	10160 ± 4541
**As**	417.3	342.4	1163	945.7	770.6	601.9	573.9	1460	773.9	1305	835.4 ± 377.0
**Sr**	287.3	255.6	235.3	183.7	231.6	242.6	219.7	201.5	131.8	188.1	217.7 ± 43.57
**Ag**	1293	1169	944.1	810.2	955.0	1025	647.3	960.4	480.0	2062	1034 ± 430.6
**Cd**	0.2610	0.3060	0.1660	0.2100	0.2110	0.2470	0.2090	0.3360	0.4590	1.595	0.4000 ± 0.4280
**Cs**	0.5050	0.4310	0.3680	0.3150	0.3980	0.3870	0.4660	0.7810	0.3450	0.4290	0.4430 ± 0.1320
**Ba**	57.96	279.2	42.44	36.19	48.28	53.62	62.67	383.4	41.55	331.8	133.7 ± 138.9
**Pb**	11690	10976	7595	6416	8875	10184	727.2	1767	1571	8341	6814 ± 4080
**Au**	1252	1209	496.8	768.3	899.2	1099	174.8	163.3	155.0	155.0	637.3 ± 462.7
**Hg**	6118	5601	6267	4397	5682	5200	12024	7944	15937	4811	7398 ± 3715

The data are means of 3 determinations. The symbol “——” means undetected or below the limit of detection.

## Data Availability

The data supporting the conclusions are included in the manuscript. The data sets used and/or analyzed during the current study are available from the corresponding author on reasonable request.
